# Acoustic Flutter Processing in the Inferior Colliculus of Awake Marmosets: Complementary Rate Coding Modulated by Acoustic Parameters

**DOI:** 10.1007/s12264-026-01587-5

**Published:** 2026-03-10

**Authors:** Siyi Bai, Xinyuan Cao, Min Xie, Guanglong Sun, Xiaohui Wang, Leilei Zheng, Xinjian Li, Zheng Lin, Lixia Gao

**Affiliations:** 1 Department of Psychiatry, The Second Affiliated Hospital and School of Brain Science and Brain Medicine, Zhejiang University School of Medicine, Hangzhou, 310058 China; 2 Nanhu Brain-computer Interface Institute, Hangzhou, 311100 China; 3 NHC and CAMS Key Laboratory of Medical Neurobiology, MOE Frontier Science Center for Brain Science and Brain-machine Integration, School of Brain Science and Brain Medicine, Zhejiang University, Hangzhou, 310058 China; 4 Key Laboratory of Biomedical Engineering of the Ministry of Education, College of Biomedical Engineering and Instrument Science, Zhejiang University, Hangzhou, 310027 China

**Keywords:** Acoustic flutter, Repetition rate, Inferior colliculus, Synchronized neurons, Non-synchronized neurons, Monotonic rate coding, Nonhuman primate, Marmoset

## Abstract

The acoustic flutter is processed through complementary monotonic rate coding and cannot be modulated by other acoustic parameters in the auditory cortex (AC). However, it remains unclear how the inferior colliculus (IC) encodes acoustic flutter, especially when changing other acoustic parameters. Here, we recorded IC neural activity in response to acoustic flutter and determined the existence of conjunctive processing between repetition rate and other acoustic parameters. We found that most IC neurons also encode the repetition rate at the flutter range through complementary monotonic rate coding. In addition, although the acoustic parameters did not change their monotonicity, most IC neurons encode both repetition rate and other acoustic parameters, different from the flutter processing in AC. Thus, complementary monotonic rate coding for acoustic flutter was widespread in the auditory system; however, coding specificity for repetition rate increased from IC to AC, and the capacity for conjunctive coding with other acoustic parameters decreased.

**Supplementary Information:**

The online version contains supplementary material available at 10.1007/s12264-026-01587-5.

## Introduction

As we know, the flutter [ranging from 10 to 45 pulses per second (pps)] broadly exists in most natural and human-made acoustic stimuli, such as aeroelastic flutter of feathers [1], the sounds of drumming, doorbell, cricket song, and so on [2, 3]. A sound is perceived as a single continuous event at a higher repetition rate than flutter, such as the pitch of the voice [2, 4, 5]. Otherwise, it is perceived as independent sound at lower repetition rates [2]. The perception of acoustic flutter is important for consonant and stress recognition, which is especially crucial for understanding sentences in human speech [3, 6, 7]. Previously, the neural mechanisms of acoustic flutter were mainly studied in the AC [2, 8], which uses both temporal periodicity (stimulus-synchronized response) and the mean firing rate (stimulus non-synchronized response) to encode acoustic flutter [2, 8, 9]. In addition, the firing rate of the auditory cortical neurons could be either increased or decreased monotonically with the increasing repetition rate at the acoustic flutter range, regardless of whether the neurons were stimulus-synchronized or non-synchronized [2, 8, 10]. These two distinct populations were proposed to encode acoustic flutter through an opponent/complementary rate coding mechanism [2, 8, 11]. Notably, the response to repetition rate at the flutter range of AC neurons was not affected by other acoustic parameters [2], indicating that the AC has distinct neuronal populations that specifically encode acoustic flutter. However, it remains unclear how subcortical regions contribute to the acoustic flutter perception.

The IC was proven to serve as a pivotal relay and processing center in the auditory pathway, which integrated inputs from the brainstem and sent refined outputs to the medial geniculate body (MGB) and the AC for further processing and perception [12–15]. Our previous study revealed that IC encodes the time-varying stimuli in a millisecond scale through both positive-monotonic and negative-monotonic rate coding [12]. This study changed the view that the strategy of complementary rate coding only existed in the AC of nonhuman primates [2, 8, 16, 17]. However, this study used a larger time scale of repetition rate (10–500 pps) with a few stimuli at the flutter range [12]. In addition, the IC comprises three primary subdivisions: the central nucleus (CNIC), the dorsal cortex (DCIC), and the external cortex (ECIC), which have distinct functions during acoustic processing. So, it is still unclear how IC encodes the repetition rate at the flutter range. In addition, acoustic flutter stimulation always includes other acoustic parameters, such as sound intensity, periodicity, and the spectral and envelope of a click [2]. These sound parameters are also processed by the IC [7, 13, 18–21]. A critical remaining question is whether and how these sound parameters modulate the flutter processing in IC. By using single-unit recordings in awake marmosets and a computational regression model, we first confirmed the existence of complementary monotonic rate coding in IC for acoustic flutter processing. Secondly, we found the conjunctive processing of different acoustic parameters of IC neurons at the flutter range. Combined with previous studies, our study indicated that complementary monotonic rate coding in response to flutter stimuli is prevalent in the acoustic system; however, the capacity of conjunctive processing between repetition rate and other acoustic parameters exhibits a progressive decline from the inferior colliculus to the auditory cortex.

## Materials and Methods

### Animal Ethics

All experiments were conducted under the guidelines of the Zhejiang University (ZJU) Committee for the Care and Use of Laboratory Animals. All the experimental protocols were approved by the Animal Advisory Committee at ZJU and followed the National Institutes of Health (NIH) guidelines. Male and female marmosets (350–450 g, 2–4 years old) were purchased from Johnbio (Jiangsu, China) and kept in pairs at the Nonhuman Primate Center at ZJU. The monkeys were housed in a colony room with a maintained temperature of 26–28°C and humidity of 45%–55% with a 12-h light/dark cycle. The marmosets were fed daily with 30–40 g of nutritious food [22, 23].

### Animal Preparation

Two adult common marmosets (Callithrix jacchus) of either sex at 2–3 years of age were used in this study. The detailed experimental procedures were described in our previous publication [12, 24]. In brief, we trained the animals to sit quietly in a marmoset chair from 15 min gradually to 2 h per day for two weeks, ensuring that electrophysiological recordings could be accomplished in awake, head-fixed animals. Head cap implantation surgery was conducted under sterile conditions, and two head posts were attached to the skull of each experimental animal for head fixation. Two recording chambers were built with dental cement over the temporal sides, and the lateral sulcus (LS) was traced as a landmark to identify the location of the IC. The animals were allowed to recover from the surgery for at least four weeks and trained again to sit quietly in a custom-made marmoset chair for electrophysiological recordings.

### General Procedures

Single-unit recordings were made *via* high-impedance tungsten microelectrodes (1–3 MΩ, FHC). The signals were acquired (AlphaLab SNR, Alpha Omega Engineering, Nof HaGalil, Israel) and transmitted (RX6, Tucker-Davis Technologies, Alachua, FL, USA), analyzed, and saved *via* custom programs written in MATLAB (MathWorks). The action potentials were detected online *via* a template-matching method (AlphaLab SNR, Alpha Omega Engineering). Acoustic stimuli were generated digitally (RX6, Tucker-Davies Technologies), attenuated (PA5, Tucker-Davies Technologies), power amplified (D75A, Crown Audio, Northridge, CA, USA), and delivered by a free-field loudspeaker (LS50, KEF, Maidstone, United Kingdom) located approximately 1 meter in front of the animals. All the recording sessions were performed in a soundproof and electromagnetic shielded chamber.

### Targeting IC Neurons

The method used to assess the IC was the same as that used in a previous study [12]. To access the IC, small craniotomies of 1 mm in diameter were made posterior to A1 in the recording chambers, allowing for the penetration of electrodes. The electrodes penetrated the brain along a dorsolateral-to-ventromedial trajectory at an angle of 45° lateral to the medial trajectory and traversed 8–10 mm of brain tissue before reaching the IC. In general, 10–25 penetrations were made through each miniature hole (Fig. S2B and 2D), which was then sealed using dental cement. After one week of rest, another miniature hole in the skull was opened for further studies.

### Acoustic Simulation

**Fig. 1:**
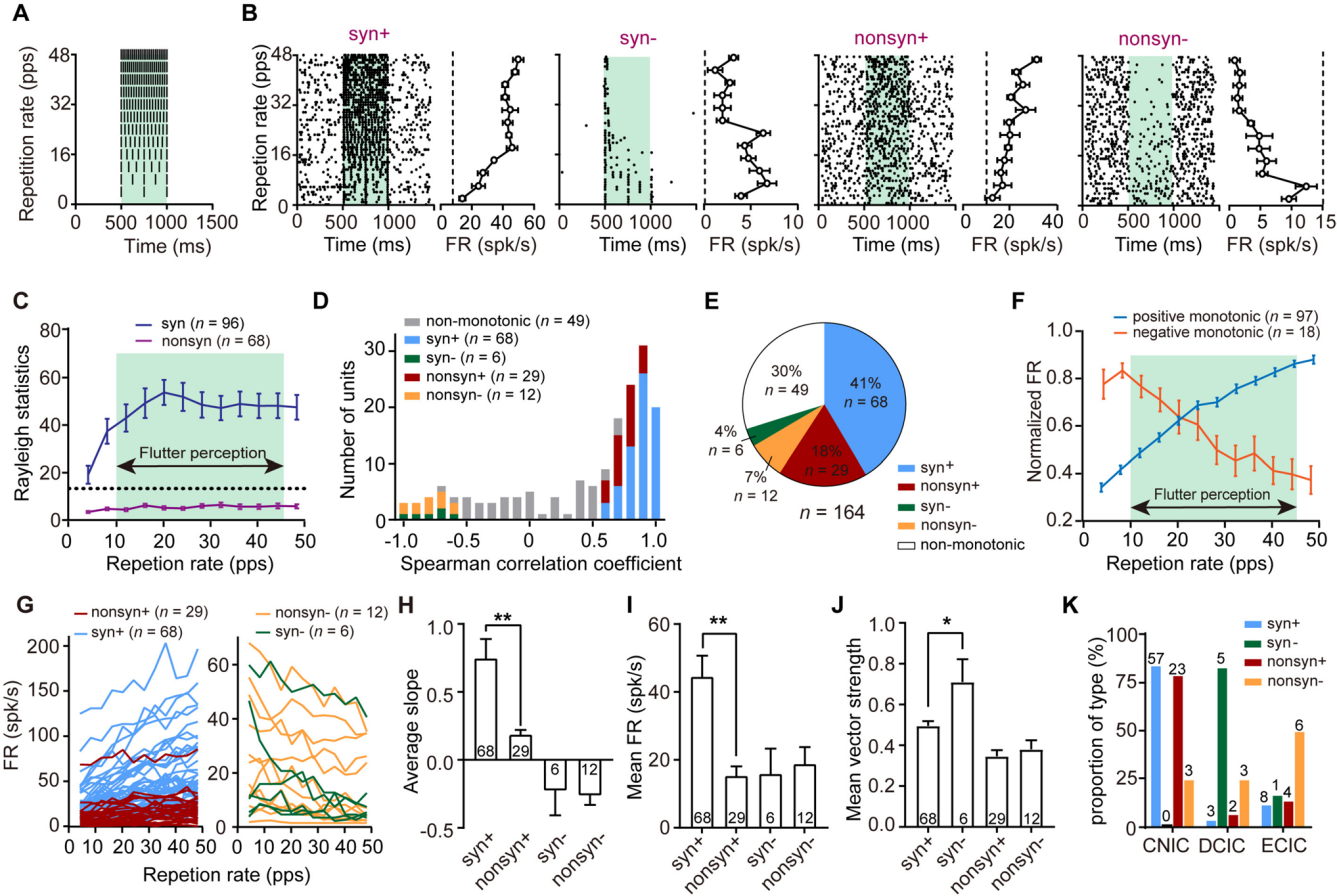
Complementary monotonic rate coding for acoustic flutter by stimulus-synchronized and stimulus non-synchronized neurons in IC. Syn+, positive-monotonic synchronized response; syn-, negative-monotonic synchronized response; nonsyn+, positive-monotonic non-synchronized response; nonsyn−, negative-monotonic non-synchronized response. **A** The diagram of acoustic pulse trains spanning the perceptual range of acoustic flutter with the repetition rate varying from 4 to 48 pps in a 4-pps step. Each vertical black line indicates a click. The green shaded area indicates the periods of acoustic stimulation, which were 500 ms. **B** Examples showing syn+, syn-, nonsyn+, and nonsyn- responses to Gaussian click trains with varying repetition rates in the flutter range. In each example, left, raster plots of the spiking response (green shading, periods of acoustic stimulation); right, corresponding firing rates averaged throughout acoustic stimulation and across trials plotted against the repetition rate. The dotted line is the averaged spontaneous firing rate. FR, firing rate. **C** The Rayleigh statistics (RS) of synchronized (syn, blue) and non-synchronized (nonsyn, purple) IC neurons in response to the click trains at the flutter range (10–45 pps). The horizontal dashed line at 13.8 indicates significance in the RS, which is equal to *P* < 0.001. **D** The distribution of the Spearman correlation coefficient (CC) for syn+, syn−, nonsyn+, and nonsyn− and non-monotonic neurons as a function of the repetition rate. Monotonic neurons have a significant CC value (*P* < 0.05 for a positive or negative correlation). Neurons with CC values of 1 and −1 indicate that they have perfect positive and negative monotonicity, respectively. **E** Proportions of IC neurons with syn+, syn−, nonsyn+, nonsyn−, and non-monotonic responses. **F** The normalized mean firing rate of positive monotonic and negative monotonic IC neurons as a function of repetition rate. The green shaded area indicates the acoustic flutter range. **G** Firing rate of individual IC neurons with positive monotonic (Left) and negative monotonic (Right) responses to different repetition rates at flutter range. **H** Mean response slope of syn+, syn−, nonsyn+, and nonsyn- neurons in IC to different repetition rates at the flutter range (syn+ and nonsyn+, *P* = 0.0048). **I** Mean firing rates during the stimulation period of syn+, syn−, nonsyn+, and nonsyn− neurons in response to different repetition rates at the flutter range (syn+ and nonsyn+, *P* = 0.0029). **J** Mean vector strength (VS) of syn+, syn−, nonsyn+, and nonsyn− neurons in IC in response to different repetition rates at the flutter range (syn+ and syn−, *P* = 0.0142). **H–J** unpaired *t* test, *P < 0.05, **P < 0.01. **K** Proportion of syn+, syn−, nonsyn+, and nonsyn− neurons in IC subdivisions. CNIC, the central nucleus of the inferior colliculus; DCIC, the dorsal cortex of the inferior colliculus; ECIC, the external cortex of the inferior colliculus. The number indicates the number of neurons.

As a single unit was isolated, its basic response properties, such as best frequency (BF) and best level (BL), were measured *via* pure tones with steps of 0.1 octave and 10 dB. We generated a series of Gaussian narrowband acoustic pulse trains (Gaussian clicks) with repetition rates ranging from 4 to 48 pps in a 4-pps step (Fig. 1A). The carrier frequency of the Gaussian click was set at the BF of the recorded neuron. Each click was generated by windowing the preferred carrier frequency *via* a Gaussian envelope. The duration of the Gaussian click train was 500 milliseconds (ms), and the duration of pre-stimulus and post-stimulus both lasted for 500 ms. To examine whether the envelope of the Gaussian click affects the coding of the acoustic flutter, we varied the standard deviation (σ) of the Gaussian click in the range of 0.1–0.4. The regularity of the Gaussian click trains was generated by temporally shifting each click within a regular pulse train by increasing the jitter of the inter-click interval (ICI) from 12.5%, 25%, 37.5%, and 50% randomly selected from a uniform distribution without affecting the average repetition rate [2]. All acoustic pulse trains with varying ICIs were presented at an inter-stimulus interval of 1 s in a randomly shuffled order and were repeated 5–10 times. The stimulus intensity of Gaussian click trains generally varies from 30–70 dB SPL.

### Data Analysis

The data were analyzed using custom programs written in MATLAB (MathWorks). The analysis methods used were the same as those in previous publications [2, 8, 12, 24, 25]. In brief, the firing rate elicited by acoustic stimulation was calculated over the entire stimulus duration, and the mean spontaneous rate was estimated over the entire stimulus set. BF was defined as the tone frequency that generated the highest firing rate. BL was defined as the sound level of the BF that generated the highest firing rate.

$$ VS = \frac{1}{n}\sqrt {\left( {\sum\limits_{1}^{n} {\cos \theta i} } \right)^{2} + \left( {\sum\limits_{1}^{n} {\sin \theta i} } \right)^{2} } $$

The synchronized spiking response was quantified by the vector strength (VS) *via* the following formula [26, 27]:

$$ {\theta i} = 2{\uppi }\frac{{{\mathrm{ti}}}}{{\mathrm{T}}} $$

Where n is the number of spikes in the analysis window, 50 ms after stimulus onset to the offset of the stimulus; ti is the time of spike occurrence; and T is the ICI. VS may vary from 0 to 1. A value of 0 implies a random relationship between acoustic stimulation and the neural firing rate. A value of 1 implies perfect synchrony between them. The significance of the synchronized discharge was statistically assessed with Rayleigh statistics (RS, 2n*VS^2^) ^[26,27]^. The Rayleigh statistic was computed for the first half and second halves of the stimulus, and the minimum value was used. This excluded some responses that were synchronized during the onset or offset of the stimulus. The values > 13.8 were equivalent to *P* < 0.001.

The monotonicity of spiking responses versus the repetition rate function was determined by the Spearman correlation coefficient (CC value, *P* < 0.05 for a positive or negative correlation). We calculated monotonicity over all repetition rates between 4 and 48 pps. For each neuron, we also performed a linear regression on neural spiking activity to repetition rates between 4 and 48 pps and determined whether the response slope was significantly different from zero (*F* test, *P* < 0.05).

Stepwise regression was used to examine whether the neural firing rate of the IC was significantly affected by different acoustic parameters of flutter sounds. The repetition rate, sound level, shape of the Gaussian click (spectral and envelope), and irregularity of the click trains were considered independent variables, which were introduced into the linear regression model one by one through the *F* test and t-tests. The mean firing rate of IC neurons was used as a dependent variable. The selection of the potential explanatory variables was determined by calculating the partial regression sum of squares of the variables introduced into the equation and performing a significance test (*t* test). If *P* < 0.05, the variable was added to the equation. If *P* > 0.05, the variable was removed from the equation. The equation of the linear regression model is shown below:

$$y=a{x}_{1}+b{x}_{2}+\varepsilon $$

Where *y* is the dependent variable, *x*_*1*_ is the first independent variable, *x*_*2*_ is the second independent variable, *a* is the regression coefficient for the first independent variable, *b* is the regression coefficient for the second independent variable, and *ꜫ* is the random error term. First, the variables *x* and *y* are introduced, the significance test is performed, and the weight coefficient is obtained. If the *F* test is greater than the critical value, the variable *x*_*1*_ is added to the equation; otherwise, it is removed, indicating that the variable *x*_*1*_ has no significant influence on the explanatory power of the dependent variable. To eliminate the influence of different dimensions of sound parameters, all independent variables were normalized by standardized z scores into standardized variables.

### Statistical Analysis

All values are expressed as the mean ± SEM unless otherwise specified. Comparisons between neural responses to acoustic flutter with two changing sound parameters were analyzed *via* two-way ANOVA. *P* < 0.05 was considered statistically significant for all the analyses and is indicated with asterisks. *P < 0.05; **P < 0.01; ***P < 0.001 and ****P < 0.0001.

## Results

### Both Synchronized and Non-synchronized IC Neurons Showed Complementary Rate Coding to Acoustic Flutter

In the present study, we recorded 205 well-isolated single units from the IC of two awake marmosets. Among them, 164 neurons exhibited significant auditory responses to the acoustic flutter stimuli. To study temporal processing at the flutter range (10–45 pps), we delivered acoustic pulse trains at repetition rate in the range of 4–48 pps that varied linearly in 4-pps step (Fig. 1A). As shown in Figure 1B, there are four types of IC neural response to acoustic flutter based on their firing rate and temporal periodicity: positive-monotonic synchronized response (syn+), negative-monotonic synchronized response (syn-), positive-monotonic non-synchronized response (nonsyn+), and negative-monotonic non-synchronized response (nonsyn−), which is consistent with previous studies in the IC examined with larger time scale of repetition rate (10–500 pps) [12] and the AC examined at the flutter range [2]. In brief, both syn+ and syn- neurons exhibited precise click-synchronized spiking activity with a vector strength (VS) > 0.1 and Rayleigh statistics (RS) > 13.8 at most or all repetition rates in the flutter range (Fig. S1A–B). In contrast, nonsyn+ and nonsyn- neurons did not show stimulus-synchronized spiking activity (Fig. S1C–D, RS < 13.8). Thus, stimulus-synchronized neurons (syn, including syn+ and syn–) could be clearly distinguished from non-synchronized neurons (nonsyn, including nonsyn+ and nonsyn–) by RS (Fig. 1C and Fig. S1). In addition, the number of syn neurons (*n* = 96) is more than the number of nonsyn neurons (*n* = 68, Fig. 1C and 1E), indicating that stimulus-synchronization is primarily used by IC neurons to encode the repetition rate of acoustic flutter. The monotonicity of the acoustic flutter-evoked response was quantified by the Spearman correlation coefficient (*R* value; see Methods). We defined neurons with *R* > 0.5 and *P* < 0.05 as positive monotonic (*n* = 97), while neurons with *R* < −0.5 and *P* < 0.05 were defined as negative monotonic (*n* = 18); the rest were defined as nonmonotonic (*n* = 49, Fig. 1D). Different from the AC having an almost equivalent proportion of positive-monotonic and negative-monotonic neurons [2], the proportion of negative monotonic neurons (11%; syn−, 4%; nonsyn−, 7%) in IC is much smaller than that of positive monotonic neurons (59%; syn+, 41%; nonsyn+, 18%, Fig. 1E).

Intriguingly, 115 out of 164 (70%) IC neurons presented either monotonic increased (positive-monotonic) or decreased (negative-monotonic) firing rates with increasing repetition rate at the flutter range (Fig.1F–1G), regardless of whether they were stimulus-synchronized or non-synchronized. Then, we fitted the temporal response of IC neurons to repetition rate at flutter range using a linear regression method and used the linear slope and mean firing rate to define their response tuning to repetition rates. Although both syn+ and nonsyn+ neurons increased firing rates with increasing repetition rates, syn+ neurons presented a larger linear fitting slope (Fig. 1H, *P =* 0.0048, unpaired *t* test) and a higher firing rate (Fig. 1I, *P =* 0.0029, unpaired *t* test) than nonsyn+ neurons. Moreover, the proportion of syn+ neurons (41%) was two times more than that of nonsyn+ neurons (18%, Fig. 1E). No significant differences in linear fitting slope (Fig. 1H, *P* = 0.8217, unpaired *t* test) and firing rate (Fig. 1I, *P* = 0.7437, unpaired *t* test) were detected between syn− and nonsyn− neurons. Interestingly, syn- exhibited better synchronization than syn+, as indicated by a larger VS (Fig. 1J, *P* = 0.0142, unpaired *t* test). Last, Syn+, syn−, nonsyn+, and nonsyn− neurons were registered into three IC subdivisions: the central nucleus (CNIC), dorsal cortex (DCIC), and external cortex (ECIC), following the same criteria as described in the previous study [12]. The majority of positive-monotonic neurons (both syn+ and nonsyn+) were predominantly localized in the CNIC (Fig. 1K). In contrast, syn-neurons primarily clustered in the DCIC, while nonsyn-neurons were mainly found in the ECIC (Fig. 1K).

### The Coding of Acoustic Flutter in IC was Modulated by Sound Intensity

**Fig. 2:**
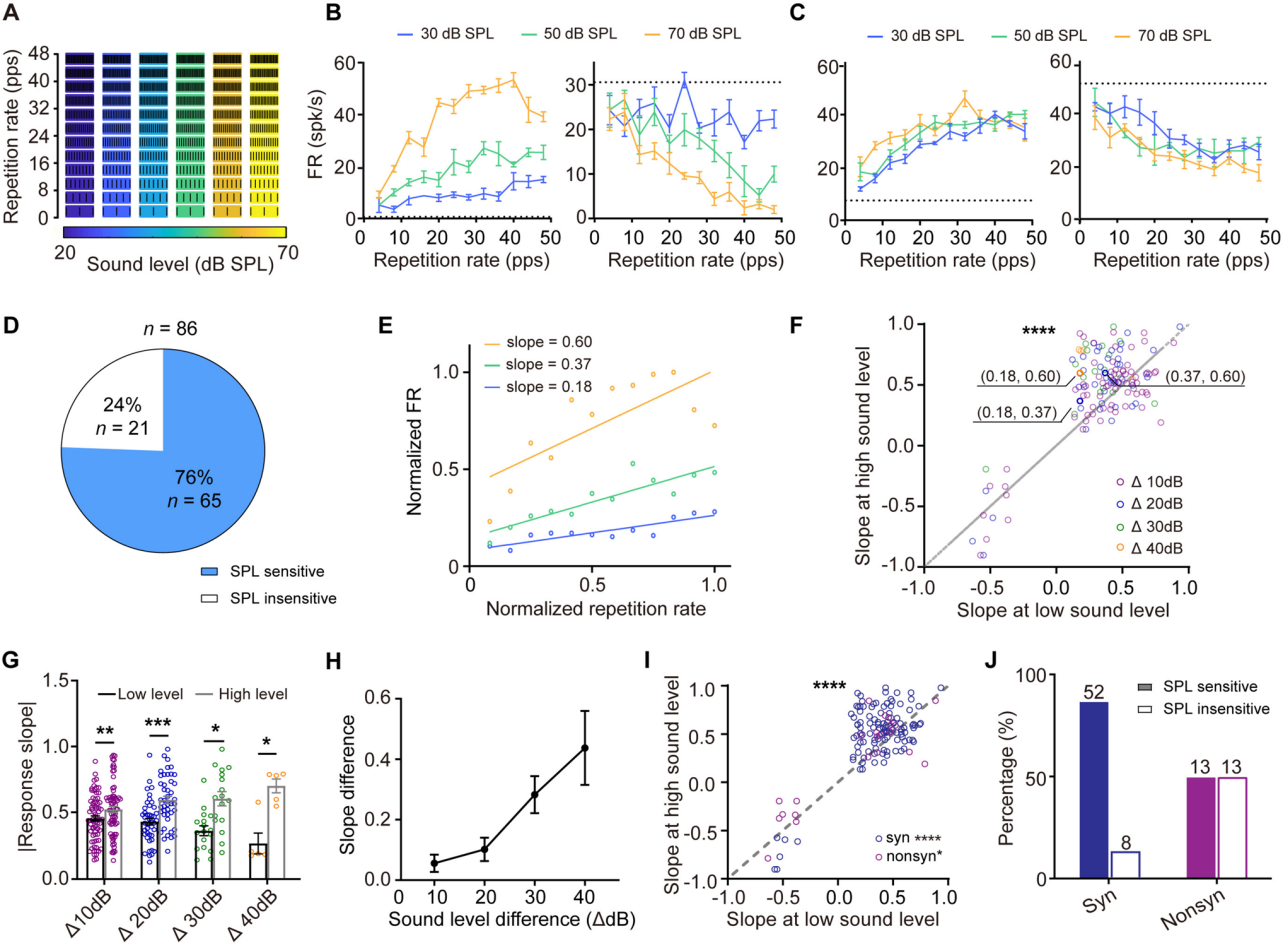
Modulation of sound intensity on IC neural coding for acoustic flutter. SPL, sound pressure level; syn, synchronized response; nonsyn, non-synchronized response.**A** Schematic showing acoustic pulse trains with a repetition rate of 4–48 pps with varying sound pressure level from 20–70 dB SPL. **B** The examples show two IC neurons that were sensitive to changes in the sound level of acoustic flutter. Left, positive-monotonic response as a function of the repetition rate. Right, negative-monotonic response as a function of the repetition rate. Blue, 30 dB SPL; green, 50 dB SPL; orange, 70 dB SPL. The dotted line indicates the averaged spontaneous firing rate. **C.** The examples show two IC neurons that were insensitive to changes in the sound level of acoustic flutter. Left, positive-monotonic response as a function of the repetition rate. Right, negative-monotonic response as a function of the repetition rate. Blue, 30 dB SPL; green, 50 dB SPL; orange, 70 dB SPL. The dotted line indicates the mean spontaneous firing rate. **D** The proportions of sound level sensitive and insensitive IC neurons encoding repetition rate at flutter range. **E** The linear regression curves of an example IC neuron in response to acoustic flutter at different sound levels (blue, 30 dB SPL; green, 50 dB SPL; orange, 70 dB SPL). **F** The linear regression slopes of IC neurons encoding acoustic flutter at low sound levels were plotted against those at high sound levels. Purple, Δ10 dB; blue, Δ20 dB; green, Δ30 dB; orange, Δ40 dB. **G** Comparison of the absolute response slopes of individual IC neurons to acoustic flutter at different sound levels. paired *t* test, *P < 0.05, **P < 0.01, and ***P < 0.001. **H** The differences in linear response slope were plotted against the differences in the sound level. **I** The linear regression slopes of IC synchronized (syn, blue) and non-synchronized (nonsyn, purple) neurons encoding acoustic flutter at low sound levels were plotted against those at high sound levels. paired *t* test, *P < 0.05, ****P < 0.0001. **J** Proportions of IC synchronized (syn, blue) and non-synchronized (nonsyn, purple) neurons, either sensitive or insensitive to the sound level of acoustic flutter.

Previous studies have demonstrated that the majority of IC neurons monotonically increased firing rates in response to the increased sound intensity [12, 21]. So, we hypothesized that the neural coding of repetition rate at the acoustic flutter range may be affected by sound intensity. To address this question, we varied the sound level of Gaussian click trains from 20 to 70 dB SPL (Fig. 2A) and recorded IC neural responses induced by repetition rates at the flutter range. We found that the monotonicity of most IC neurons to repetition rates at the acoustic flutter range was not affected by the change of the sound intensity (Fig. 2B–2C). However, the tuning curve in response to repetition rate of some IC neurons was affected by sound intensity (Fig. 2B), and some not (Fig. 2C). Using two-way ANOVA analysis, we found that 76% of IC neurons exhibited a significantly altered tuning in response to repetition rate with varying sound levels (Fig. 2D). These results were different from those reported in the AC, that the firing rates were largely unaffected by the change of sound level [2]. To further examine how the encoding of the repetition rate at flutter range was modulated by the sound intensity, we calculated the linear fitting slope of the IC neurons in response to the repetition rate at different sound intensities (Fig. 2E, see Methods). We found that the response slopes at higher SPL were significantly greater than those at lower SPL (Fig. 2F, *P* < 0.0001; paired *t* test; Fig. 2G, ∆10 dB, *P =* 0.0023; ∆20 dB, *P =* 0.0003; ∆30 dB, *P =* 0.0339; ∆40 dB, *P =* 0.0214, paired *t* test). In addition, the slope difference increased with the difference between the sound intensity (Fig. 2H, *P =* 0.0014, *F* = 5.441; one-way ANOVA). Last, we examined whether syn and nonsyn neurons were affected differently by sound intensity (Fig. 2I) and found that syn neurons were affected more than nonsyn neurons by the change of sound intensity (Fig. 2I, syn, *P* < 0.0001; paired *t* test; nonsyn, *P* < 0.05; paired *t* test). In contrast to a similar percentage of SPL-sensitive and SPL-insensitive nonsyn neurons, a larger proportion of syn neurons were sensitive to the changes of sound intensity (Fig. 2J).

**Fig. 3:**
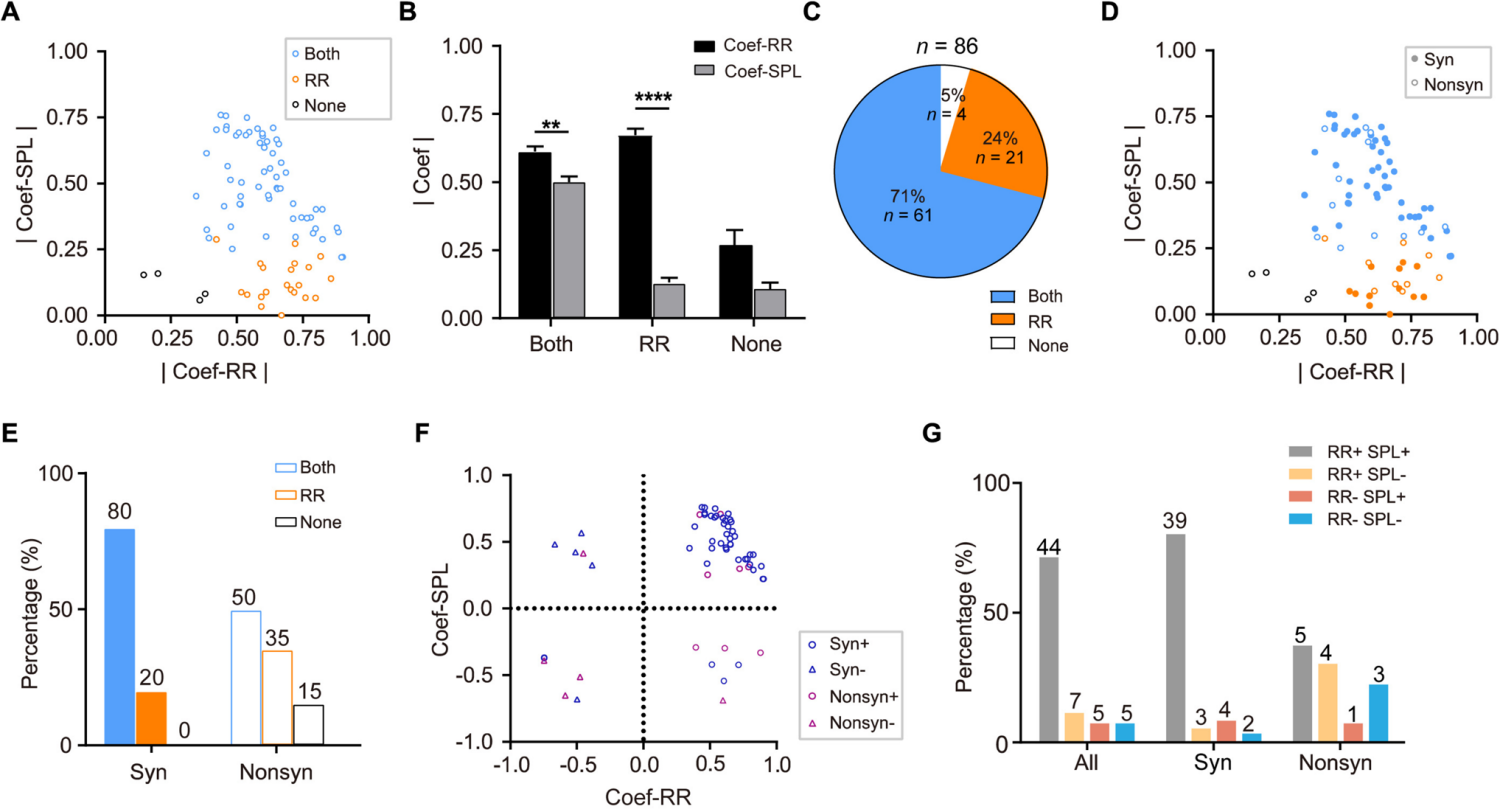
The weight of repetition rate and sound intensity on the IC neural discharge to acoustic flutter. **A** The distribution and classification of Both-type, RR-type, and None-type neurons according to the absolute values of the RR coefficient (Coef-RR) and SPL coefficient (Coef-SPL), which were calculated through a stepwise regression model. Blue, Both-type neurons sensitive to both repetition rate and sound intensity; Orange, RR-type neurons only sensitive to repetition rate; Black, None-type neurons insensitive to both repetition rate and sound intensity. **B** The comparison of the absolute value of the RR coefficient and SPL coefficient of Both-type, RR-type, and None-type neurons. **P < 0.01, ****P < 0.0001. **C** The proportions of Both-type, RR-type, and None-type neurons. **D** The distribution and classification of IC neurons with syn (filled) and nonsyn (empty) responses, respectively, according to the absolute values of the RR coefficient and SPL coefficient, which were calculated through a stepwise regression model. **E** The proportions of Both-type, RR-type, and None-type neurons in IC syn and nonsyn populations. The number indicates the number of neurons. **F** The distribution and classification of syn+; syn−; nonsyn+ and nonsyn− neurons, respectively, according to the values of the RR coefficient and SPL coefficient. **G** Proportions of RR+SPL+, RR+SPL−, RR−SPL+, and RR−SPL− types in IC neurons (All) or those with syn and nonsyn responses. The number indicates the number of neurons. RR+SPL+: Positive monotonicity to both repetition rate and sound pressure level; RR+SPL−: Positive monotonicity to repetition rate and negative monotonicity to sound pressure level; RR−SPL+: Negative monotonicity to repetition rate and positive monotonicity to sound pressure level; RR−SPL−: Negative monotonicity to both parameters.

Then, we applied stepwise regression, a step-by-step iterative construction of a linear regression model, to examine whether the firing rate of the IC neurons was affected by the repetition rate and sound intensity of the acoustic pulse trains (see Methods). Repetition rate was introduced into the model as the first independent variable (*x*_*1*_), and the mean firing rate of IC neurons in response to repetition rate was used as a dependent variable (*y*). The regression coefficient (*a*) was obtained to represent the coding weight of IC neurons to repetition rate, which is named Coef-RR. To validate the regression model in repetition rate-related acoustic flutter processing, we tested this model using positive-monotonic or negative-monotonic neurons defined by Spearman correlation coefficient as shown in Fig. 1D. We found that 82 out of 86 neurons (95.3%) met the significance criteria, indicating that the computational model is highly reliable in quantifying the relative contributions of acoustic parameters in acoustic flutter processing. Subsequently, SPL was introduced into the model as the second independent variable (*X*_*2*_) after the repetition rate was introduced. The Coef-SPL represented the weight of SPL in acoustic flutter processing. In this model, we obtained both Coef-RR and Coef-SPL of all examined IC neurons, which represented the weights of IC neurons in encoding the repetition rate and sound intensity. An IC neuron was defined as an RR-type neuron if its firing rate was significantly affected by repetition rate only; it was defined as Both-type neuron if the firing rate was affected both by repetition rate and sound intensity; otherwise, it was defined as a None-type neuron (Fig. 3A). We found that the three types of neurons were separated and clustered in a two-dimensional space using absolute Coef-RR and Coef-SPL (Fig. 3A). None-type neurons had small absolute values of Coef-RR and Coef-SPL, which accounted for a very small proportion (Fig. 3A–3C, 5%, *n* = 4). Interestingly, Both-type neurons accounted for the largest proportion (Fig. 3C, n = 61) and had significantly larger absolute values of Coef-RR than Coef-SPL (Fig. 3B, P < 0.01, paired *t* test), suggesting that Both-type neurons had a coding bias to the repetition rate. Next, we calculated the Coef-RR and Coef-SPL of syn and nonsyn neurons separately (Fig. 3D) and found that all the None-type neurons were nonsyn neurons (Fig. 3E). The proportions of RR-type and Both-type neurons in the non-synchronized population were similar; in contrast, the number of Both-type neurons in the syn population was four times greater than that of RR-type neurons (Fig. 3E).

Last, to test how sound intensity is correlated with the monotonicity of IC neurons in processing of the repetition rate, we calculated the Coef-RR and Coef-SPL of syn+, syn−, nonsyn+, and nonsyn− neurons separately and used a 2-dimensional space to illustrate their correlation (Fig. 3F, only for both-type neurons). We found that the majority of neurons (72%) were located in the first quadrant, indicating the positive monotonic encoding of both repetition rate and sound intensity (Fig. 3F–3G). However, the remaining neurons (28%) were distributed in the other three quadrants (Fig. 3F). After separating syn neurons from nonsyn neurons, we found that most syn neurons clustered in the first quadrant, whereas nonsyn neurons were evenly distributed across all quadrants (Fig. 3F–3G).

### The Neural Coding of Acoustic Flutter was Affected by the Click Shape

**Fig. 4:**
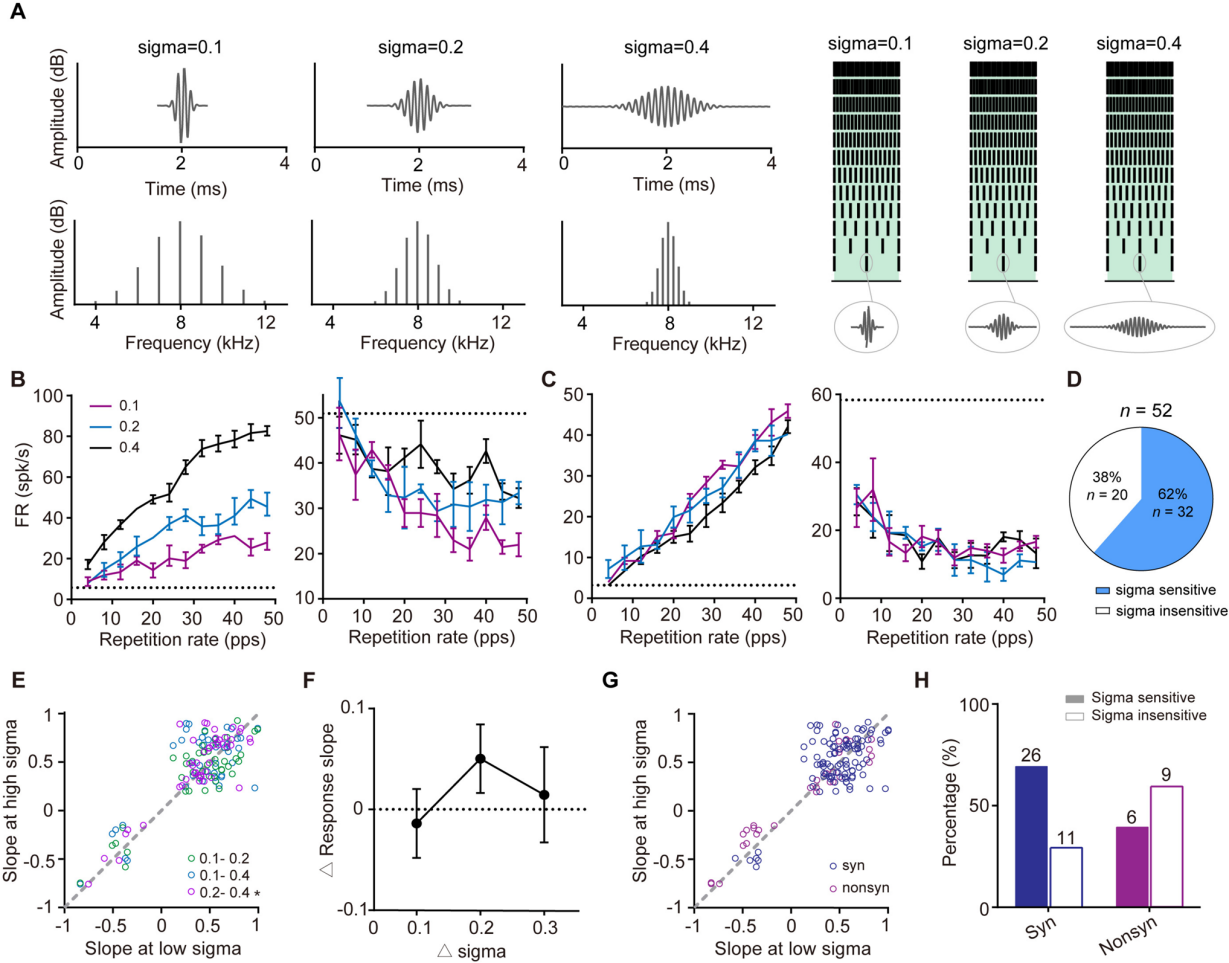
The modulation of sigma on acoustic flutter-evoked response in IC neurons. Syn, synchronized response; nonsyn, non-synchronized response. **A** Left, the acoustic waveform (top) and spectrum (bottom) of a Gaussian click with different sigma (σ) values (0.1, 0.2, 0.4). The sigma determines the envelope and spectral component of each click. Right, acoustic flutter with varying repetition rate and sigma. Each vertical black line indicates a click. The green shaded areas indicate periods of acoustic stimulation (500 ms). **B** Two representative IC neurons sensitive to both repetition rate and sigma of acoustic flutter stimulation. Left, positive monotonic neuron; Right, negative monotonic neuron. The dotted line indicates the mean spontaneous firing rate. **C** Two representative IC neurons sensitive to repetition rate at flutter range, but not to the sigma of the Gaussian-click train. **D** Proportions of σ-sensitive and σ-insensitive IC neurons encoding repetition rate at flutter range. **E** The linear regression slopes of the IC neurons in response to repetition rate at low σ were plotted against those at high σ. Different colors represent different σ gradients. paired *t* test, *P < 0.05. **F** The difference in the linear response slope of IC neurons in response to acoustic flutter plotted against different σ gradients. **G** The linear regression slopes of the IC neurons in response to repetition rate at low σ were plotted against those at high σ. Different colors represent syn (blue) or nonsyn (purple) neurons. **H** The proportions of syn and nonsyn IC neurons are either sensitive or insensitive to the σ of the Gaussian click. The number indicates the number of neurons.

To examine whether the IC neural coding of repetition rate in the acoustic flutter range is affected by the spectral and envelope of the Gaussian click, we varied the standard deviation (σ, sigma) of the Gaussian click to alter the frequency bandwidth and envelope of the individual click (Fig. 4A). We found that the monotonicity of most IC neurons in response to the repetition rate of acoustic flutter was also not affected by the click shape (Fig. 4B–4C). However, the tuning curve of some IC neurons to repetition rate was affected by click shape (Fig. 4B), whereas the others were not (Fig. 3C). Quantitative analyses revealed that 62% of IC neurons significantly altered their tuning curves as a function of the repetition rate in the flutter range, characterized by a higher firing rate with larger sigma values. In contrast, the remaining 38% of IC neurons showed no significant changes in their tuning properties (Fig. 4D). Next, we calculated the linear slope for individual IC neurons encoding acoustic flutter when sigma was 0.1, 0.2, and 0.4 (Fig. 4E–4F). We found no overall significant change of response slope with altered sigma (Fig. 4E–4F, 4F, *P* = 0.8232,* F* = 0.1948; one-way ANOVA). Then, we separately examined whether syn and nonsyn neurons were affected differently by sigma change (Fig. 4G). No significant difference was found (Fig. 4F, *P* = 0.8232, *F* = 0.1948; one-way ANOVA). Last, a larger proportion of syn in contrast to a smaller proportion of nonsyn neurons were sensitive to changes in sigma (Fig. 4H).

**Fig. 5:**
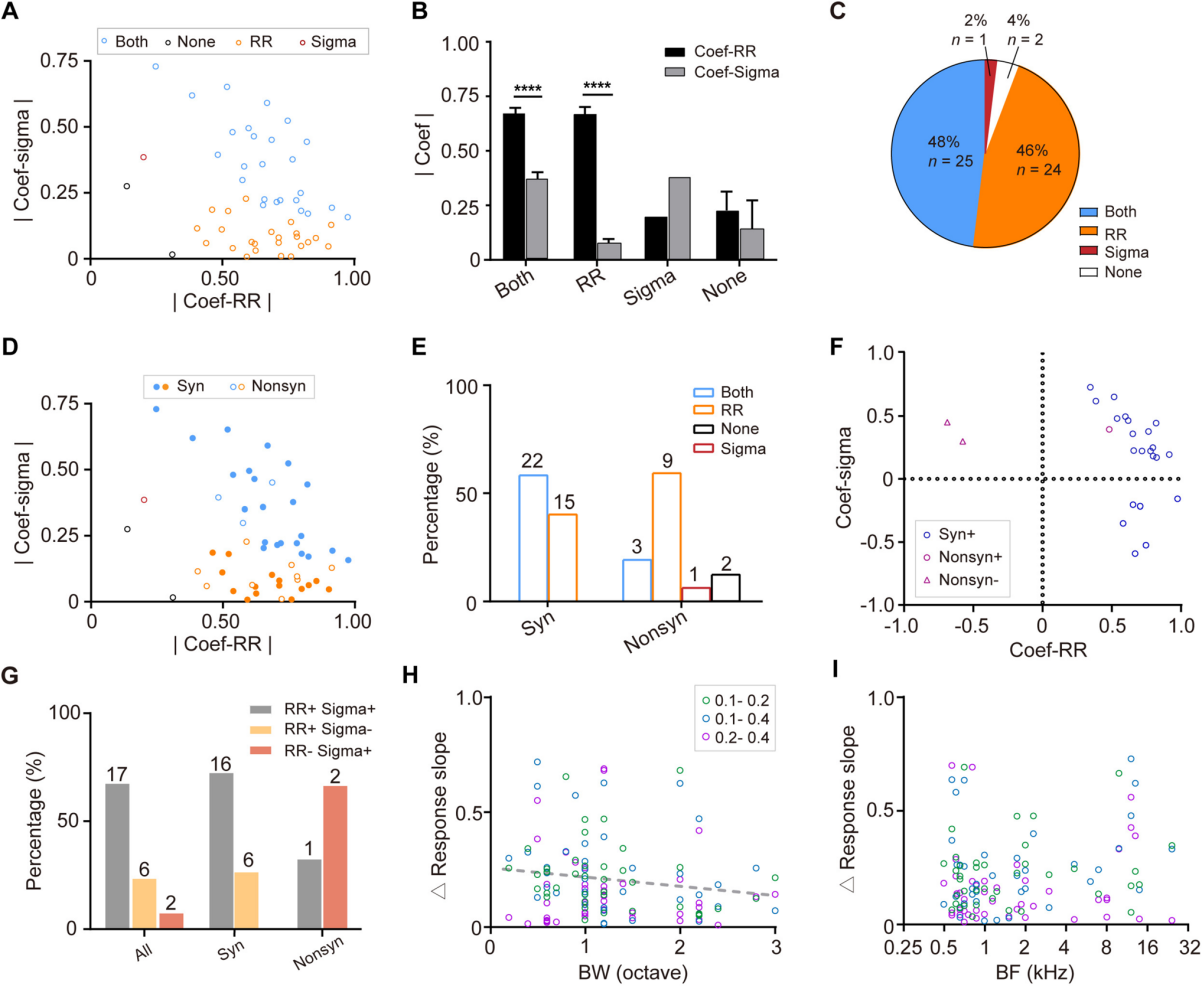
The weight of the repetition rate and sigma of the Gaussian click on the IC discharge. Syn, synchronized response; nonsyn, non-synchronized response; syn+, positive-monotonic synchronized response; nonsyn+, positive-monotonic non-synchronized response; nonsyn−, negative-monotonic non-synchronized response. **A** The distribution and classification of IC neurons according to the absolute values of the (RR) coefficient (Coef-RR) and sigma coefficient (Coef-Sigma), which were calculated through a stepwise regression model. Blue, Both-type neurons sensitive to both repetition rate and Gaussian-click σ; Orange, RR-type neurons only sensitive to repetition rate; Red brown, the Sigma-type neurons only sensitive to Gaussian-click σ; Black, None-type neurons insensitive to both repetition rate and Gaussian-click σ. **B** The absolute value of RR coefficient and sigma coefficient in Both-type, RR-type, sigma-type, and None-type neurons. ****P < 0.0001. **C** The proportions of Both-type, RR-type, sigma-type, and None-type neurons. **D** The distribution and classification of syn (filled) and nonsyn (empty) IC neurons based on the absolute value of the RR coefficient and sigma coefficient. **E** The proportions of Both-type, RR-type, sigma-type, and None-type neurons in syn and nonsyn IC populations. The number indicates the number of neurons. **F** The distribution and classification of syn+, nonsyn+, and nonsyn- IC neurons according to the RR coefficient and sigma coefficient, which were calculated through a stepwise regression model. **G** The proportions of RR+Sigma+, RR+Sigma−, and RR−Sigma+ types in IC neurons (All) or those with syn and nonsyn responses. The number indicates the number of neurons. RR+Sigma+: Positive monotonicity to both repetition rate and Sigma; RR+Sigma−: Positive monotonicity to repetition rate and negative monotonicity to sigma; RR−Sigma+: Negative monotonicity to repetition rate and positive monotonicity to sigma. **H–I** The difference of linear response slope of IC neurons to acoustic flutter plotted against the tuning bandwidth (BW, **H**) or best frequency (BF, **I**) of IC neurons. Different colors represent different σ gradients.

To further quantify how the repetition rate and sigma (σ) of a click affect the firing rate of the IC neurons, the stepwise regression model was used to calculate Coef-RR and Coef-Sigma for IC neurons. The IC neurons were classified into four groups: RR-type, Both-type, None-type, and Sigma-type neurons (Fig. 5A–5B). Similarly, an IC neuron was defined as RR-type if its firing rate was significantly affected by change of repetition rate only; an IC neuron was defined as Sigma-type if its firing rate was significantly affected by sigma change only; an IC neuron was defined as Both-type if the neural firing rate was affected not only by repetition rate but also by sigma change; otherwise, it was defined as a None-type neuron. We found that most IC neurons examined belong to the Both-type or RR-type neurons (Fig. 5C). Both-type neurons had significantly larger absolute values of Coef-RR than Coef-sigma (Fig. 5A–5B, 5B, P < 0.0001, paired *t* test). So, although the IC neural coding of repetition rate at the acoustic flutter range could be modulated by sigma, the coding bias is to represent the repetition rate over sigma. Next, we calculated the Coef-RR or Coef-Sigma of syn and nonsyn neurons separately (Fig. 5D) and found that syn and nonsyn neurons are spatially mixed. However, most of Both-type neurons were syn neurons, in contrast to similar percentage of syn and nonsyn neurons in RR-type; and all Sigma-type and None-type neurons are nonsyn neurons (Fig. 5E). Last, we further analyzed the monotonicity of Both-type neurons and plotted the Coef-RR against Coef-sigma (Fig. 5F). We found that most of Both-type neurons were distributed in the first quadrant, demonstrating a positive correlation between Coef-RR and Coef-sigma (Fig. 5F). We calculated the proportion of neurons with RR+ Sigma+; RR+ Sigma−; RR− Sigma+; RR− Sigma− in syn and nonsyn populations (Fig. 5G). We found that most syn neurons were positively modulated both by the repetition rate and Sigma (RR+ Sigma+, 72.7%, *n* = 16, Fig. 5G). In contrast, most nonsynaptic neurons were negatively modulated by the repetition rate, but positively modulated by the Sigma (RR− Sigma+, 66.7%, *n* = 2, Fig. 5G).

To examine whether the IC neural coding of sigma in acoustic flutter was correlated with their frequency tuning properties, we plotted the difference of response slope induced by sigma change against the half tuning bandwidth (BW, Fig. 5H) and best frequency (BF, Fig. 5I) of IC neurons. We found that IC neurons with narrow tuning bandwidths were more sensitive to sigma change, exhibiting a negative correlation between the difference of response slope and tuning bandwidth (Fig. 5H, Spearman correlation coefficient, *r* = −0.1771, *P* = 0.0399); however, it was not correlated with the IC neurons’ BFs (Fig. 5I, Spearman correlation coefficient, *r* = −0.0799, *P* = 0.3659). These results indicate that the neural coding of acoustic flutter was affected by the frequency tuning properties of IC neurons. The narrower the tuning bandwidth, the stronger the influence exerted by the Gaussian click on the IC neural response.

### The IC Neural Coding of Acoustic Flutter was Affected by the Irregularity of the Click

**Fig. 6:**
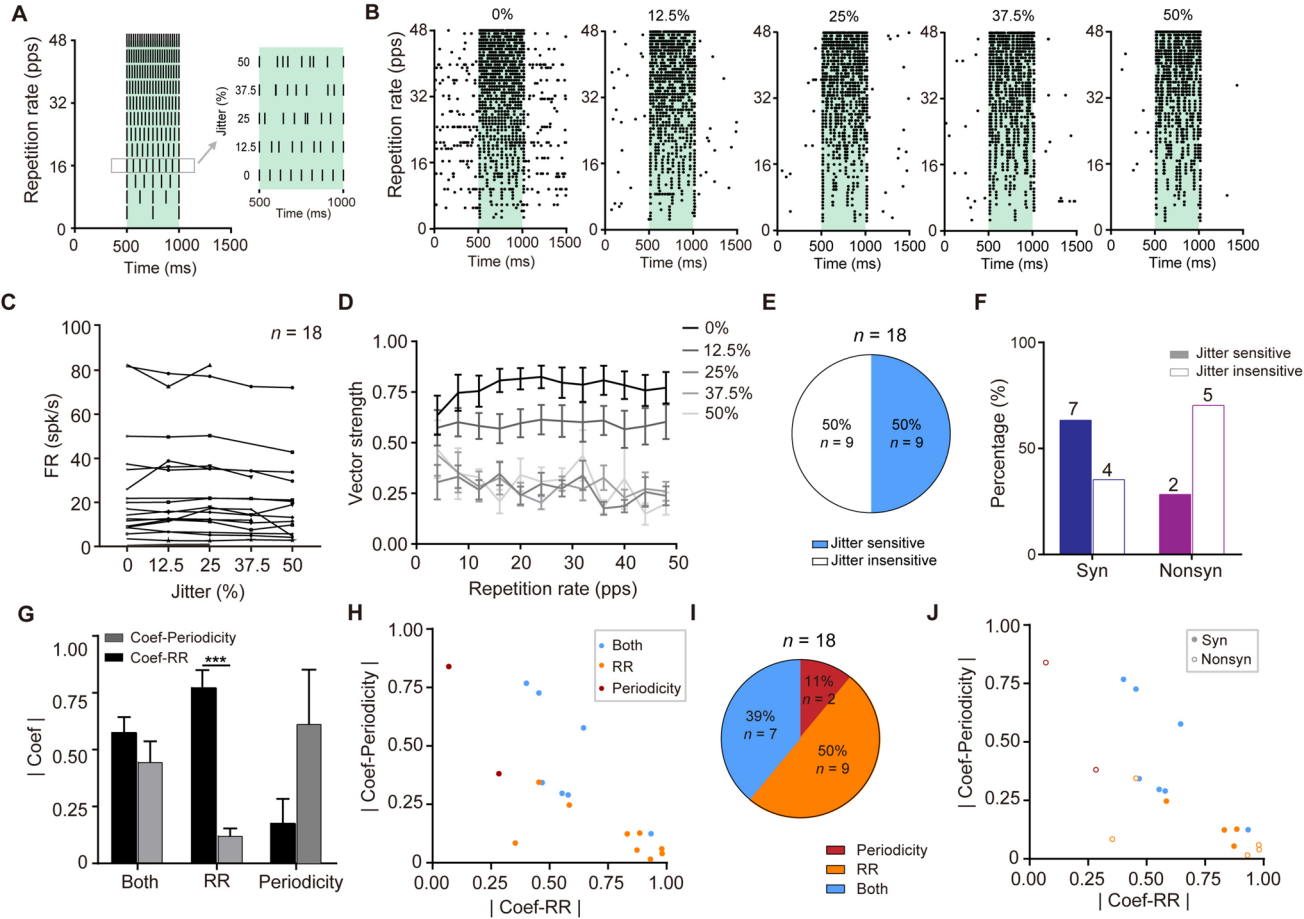
The modulation of irregularity on the acoustic flutter evoked response in IC neurons. Syn, synchronized response; nonsyn, non-synchronized response.**A** Left, the schematic showing regular acoustic pulse trains spanning the range of acoustic flutter (10–45 pps) with a repetition rate varying from 4–48 pps; Right, enlarged diagrams showing irregular acoustic pulse trains with increased irregularity (Jitter: 12.5%, 25%, 37.5%, 50%). The irregular pulse train was generated by temporally shifting the time of each click in a regular pulse train, which does not affect the average repetition rate. The repetition rate of the example Gaussian click train is 16 pps. Each vertical black line indicates a single acoustic click. The green shaded areas indicate periods of acoustic stimulation, which were 500 ms. **B** The raster plots showing an example IC neuron in response to the repetition rate of acoustic flutter with increasing degree of irregularity (from left to right, the jitter is 0, 12.5%, 25%, 37.5%, and 50%). The green shaded area indicates the range of acoustic flutter stimulation. **C** The mean firing rates of IC neurons in response to acoustic flutter as a function of changing jitter during acoustic flutter. **D** The mean VS of IC neurons was plotted against the repetition rate with different jitters. **E** The proportion of jitter-sensitive and jitter-insensitive IC neurons encoding the repetition rate of acoustic flutter. **F** The proportions of jitter-sensitive and insensitive syn (blue) and nonsyn (purple) IC neurons. The number indicates the number of neurons. **G** The averaged coef-RR and coef-periodicity among different types of IC neurons. ***P < 0.001. **H** The distribution and classification of IC neurons according to the absolute value of the RR coefficient (coef-RR) and periodicity coefficient (coef-periodicity). **I** The proportions of IC neurons sensitive to both RR and jitter (both-type), only sensitive to RR (RR-type), and insensitive to both RR and jitter (none-type). **J** The distribution and classification of syn (filled) and nonsyn (empty) IC neurons according to the absolute values of the RR coefficient and periodicity coefficient.

To examine whether the IC neural coding of repetition rate in the flutter range is affected by the temporal irregularity of acoustic stimuli, we added jitter (0, 12.5%, 25%, 37.5% and 50%) into the acoustic pulse train to increase temporal irregularity (Fig. 6A). As shown by the example neuron, syn IC neurons exhibited a phase-locked response to click even when the jitter was added to acoustic pulse trains (Fig. 6B). However, the overall firing rates of some IC neurons were affected by the temporal irregularity whereas some were not (Fig. 6C). To quantify how temporal irregularity influence IC neurons’ phase-locking responses, we employed vector strength (VS) to measure the synchronization ability of IC syn neurons in response to the repetition rate with different degrees of jitter (Fig. 6D). The value of VS significantly reduced when 12.5%, 25%, 37.5% and 50% of Jitter were added to flutter stimulation (*P* < 0.001, one-way ANOVA). The results demonstrated a progressive decline in the neurons’ ability to synchronize with individual clicks of acoustic flutter as jitter increased. Using two-way ANOVA, we found that 50% IC neurons significantly changed their firing rate in response to the increase of jitter, whereas the firing rate of the remaining neurons did not change (Fig. 6E). Among them, 63.6% of syn neurons (*n* = 7) were sensitive to jitter, and a much smaller proportion of the nonsyn neurons (28.6%, *n* = 2) were sensitive to jitter (Fig. 6F). To further examine the coding specificity of IC neurons to repetition rate and periodicity, a stepwise regression model was applied. By using this model, we obtained both Coef-RR and Coef-periodicity of all examined IC neurons. The IC neurons in this dataset were classified into three types based on a set of significance tests in the model: RR-type, Both-type, and Periodicity-type neurons. In addition to RR-type neurons, an IC neuron was defined as a Periodicity-type neuron if its firing rate was significantly affected by temporal irregularity (jitter) only, and an IC neuron was defined as a Both-type neuron if the neural firing rate was affected not only by repetition rate but also by temporal irregularity (Fig. 6G). We plotted the absolute values of Coef-RR against those of Coef-periodicity for the three groups of I C neurons (Fig. 6H). Half of the examined IC neurons were RR-type neurons, 35% were Both-type neurons, and the rest were Periodicity-type neurons (Fig. 6I). Last, we compared the Coef-RR and Coef-periodicity for syn and nonsyn neurons (Fig. 6J). We found that all the Both-type neurons were syn neurons while all the Periodicity-type neurons were nonsyn neurons (Fig. 6J). These results were different from those of a previous AC study, showing that the firing rate of AC neurons was not affected by the temporal irregularity of acoustic pulse trains in the flutter range [2].

## Discussion

In the present study, we recorded single-unit activity of IC neurons in awake marmosets and applied a linear regression model to investigate two key questions: (1) how the IC neurons encode the repetition rate in acoustic flutter range; (2) whether the encoding to repetition rate of acoustic flutter could be modulated by other acoustic parameters, such as sound intensity, irregularity, spectral, and envelope of click shape. We found that IC encodes the repetition rate in the range of acoustic flutter through complementary monotonic rate codes by both stimulus-synchronized and non-synchronized neurons, which is similar to that in AC [2]. However, IC neurons encode not only the repetition rate but also other acoustic parameters, which are distinct from the flutter processing in AC. Combined with previous studies [2, 12], the current study revealed that the rate coding, especially complementary monotonic rate coding for acoustic flutter, may be widespread in the auditory pathway; however, coding specificity for repetition rate increased from the IC to the AC, while the capacity for conjunctive coding of other acoustic parameters gradually decreased. A similar opponent rate coding was also found in the somatosensory cortex of awake and behaving macaque monkeys [16, 17], suggesting that the general coding principle is shared in different sensory systems for flutter processing.

### IC is a Processing Hub for Various Acoustic Parameters, Including Repetition Rate

The IC serves as a central hub for auditory processing, which has intrinsic networks along the acoustic pathway [13, 15, 28]. For the direct auditory pathway, the IC directly receives inputs from multiple auditory brainstem nuclei, including the cochlear nuclei, which provide tonotopically organized information about sound frequency, and the superior olivary complex, which contributes binaural cues for sound localization [29, 30]. The IC sends the ascending output signal passing through the thalamic relay station (MGB) to the AC, which is the last auditory station for sound processing [31]. Besides the direct auditory pathway, the IC receives feedback projections from the AC, enabling top-down modulation of auditory processing and sends descending outputs to lower brainstem nuclei, such as the superior olivary complex and cochlear nuclei, modulating auditory processing at earlier stages [28, 32].

Previous studies have demonstrated that different acoustic parameters, such as the sound frequency [33, 34], location [33], intensity [18], the envelope [7, 33], repetition rate [12, 19–21], and the regularity [19], are processed by the IC before transfer to higher auditory relay stations. However, few studies have addressed how IC encodes time-varying acoustic stimulation in the flutter range and whether the coding could be modulated by other acoustic parameters. In the present study, we found that IC neurons use both stimulus-synchronized and non-synchronized responses to encode Gaussian click trains with repetition rates at the flutter range. Notably, temporal processing at the millisecond scale in IC remains unchanged after AC inactivation, suggesting that it is not modulated by cortical feedback [12]. We further confirmed the existence of complementary monotonic rate coding in the IC, indicating that this coding mechanism for acoustic flutter is ubiquitous in the auditory system. Combined with previous studies [2, 12], the proportion of stimulus-synchronized responses decreases while that of non-synchronized responses increases from the IC to the AC, demonstrating a temporal-to-rate transformation along the ascending auditory pathway. This transformation may be driven by the increase of inhibitory neurons at the cortical level, which could alter the strength of inhibitory inputs to individual cortical neurons, thereby shifting the coding mode from stimulus-synchronization to firing rate coding [8].

### Complementary Monotonic Rate Coding is Crucial for Flutter Processing in the Auditory System

Stimulus-synchronized temporal coding is generally considered more precise than rate coding [10]. However, the ability of an auditory neuron to synchronize to an acoustic stimulation has an upper limit (threshold), so that the firing of a neuron could not synchronize to the repetition rate when it is over the threshold. Interestingly, the auditory system uses another strategy, which increases or decreases the neural firing following the increase of repetition rate, which is called complementary monotonic rate coding [2]. More importantly, several previous studies found that firing rate-based monotonic responses more closely match monkeys’ behavior than phase-locked temporal responses when the monkey performed a tactile or auditory frequency discrimination task [9, 16]. So, monotonic rate coding may be an evolutionary advantage for further processing temporal information. In the present study, we found that 58.5% (96/164) of IC neurons encode the repetition rate in the flutter range with the stimulus-synchronized response, while 41.5% (68/164) of IC neurons encode the repetition rate through unsynchronized responses (Fig. 1C). In addition, these neurons could be separated into positive-monotonic and negative-monotonic neurons (Fig. 1D–G), which suggests that complementary monotonic rate coding may originate as early as in the I C of the auditory system**.**

### Conjunctive Processing of Repetition Rate and Other Acoustic Parameters Embedded in Flutter

Except for the repetition rate, Gaussian click trains also contain other acoustic parameters, such as sound intensity, spectrum, and envelope of the click, and regularity of click intervals. All these acoustic parameters are important for sound perception and recognition. However, it is still unclear how IC compromises these sound parameters during acoustic flutter processing. In the present study, we found that IC neurons encoding acoustic flutter were largely affected by the sound level, spectral, and envelope of the click, as well as the irregularity of the click intervals (Figs. 2–6). In contrast to the AC, the neural coding to repetition rate in the flutter range was not affected by the sound intensity and temporal irregularity of the Gaussian click train [2]. Therefore, we conclude that the IC conjunctively processes the repetition rate and other acoustic parameters; however, the AC does not. So, the stimulus selectivity of auditory neurons progressively increases along the ascending auditory pathway [35, 36]. As we know, acoustic flutter perception is prevalent in humans, such as aeroelastic flutter of feathers, the sounds of drumming, doorbell, cricket song, and even in the pitch of different calls. The importance of flutter perception may induce a ubiquitous coding strategy along the auditory pathway.

### Prevalence of Complementary Rate Coding to Different Stimulus Features in Sensory Systems

The phenomenon of flicker or flutter is pervasive throughout all sensory systems, including visual, auditory, somatosensory, olfactory, and gustatory [3, 9, 16, 37–40]. Previous studies in the visual cortex have shown that flickering stimuli elicit oscillatory activity at the same frequency as the frequency of the visual flicker and its harmonic frequencies [41–43]. In the somatosensory system, differential neural representations of tactile stimulation were found in the peripheral and central somatosensory systems [44–47]. In the periphery, flutter sensation is mediated by primary afferent fibers with exquisitely timed, stimulus-driven spikes [46, 47]. In the primary somatosensory cortex, tactile vibration frequencies are encoded by two different neural populations: rapidly adapting neurons encode tactile stimuli in the flutter range, and Pacinian neurons encode those in higher frequencies [45, 48]. The neural encoding boundary between these two populations matches the behaviorally perceptual boundary between flutter and fusion percepts [5, 45, 49]. In addition, the neural firing rate seems to be more important for encoding of flutter frequency than temporal periodicity [17]. Importantly, complementary monotonic rate coding has been observed in the secondary somatosensory cortex of behaving monkeys [50], the AC of awake passive listening marmosets [2] and the IC of awake marmosets in the current study (Fig. 1). The opponent monotonic rate coding is generated not only by syn neurons but also by nonsyn neurons both in AC [2, 8] an IC [12]. Similar coding strategies have also been found for coding the sound level [51] and sound locations [52]. Therefore, complementary firing rate coding may be a common coding principle in the brain for encoding sensory features on the basis of differences in firing rates across neural populations [2, 12].

## Supplementary Information

Below is the link to the electronic supplementary material.

Supplementary file1 (PDF 304 KB)
