# Supplementary material for: Acoustic Flutter Processing in the Inferior Colliculus of Awake Marmosets: Complementary Rate Coding Modulated by Acoustic Parameters
Source: Neurosci Bull. 2026 Mar 10;42(8):1740–54. doi: 10.1007/s12264-026-01587-5 (PMC13424054; doi:10.1007/s12264-026-01587-5)
Supplement: Supplementary file 1 — Supplementary file1 (PDF 304 KB) [file 12264_2026_1587_MOESM1_ESM.pdf]

# Supplementary Figures

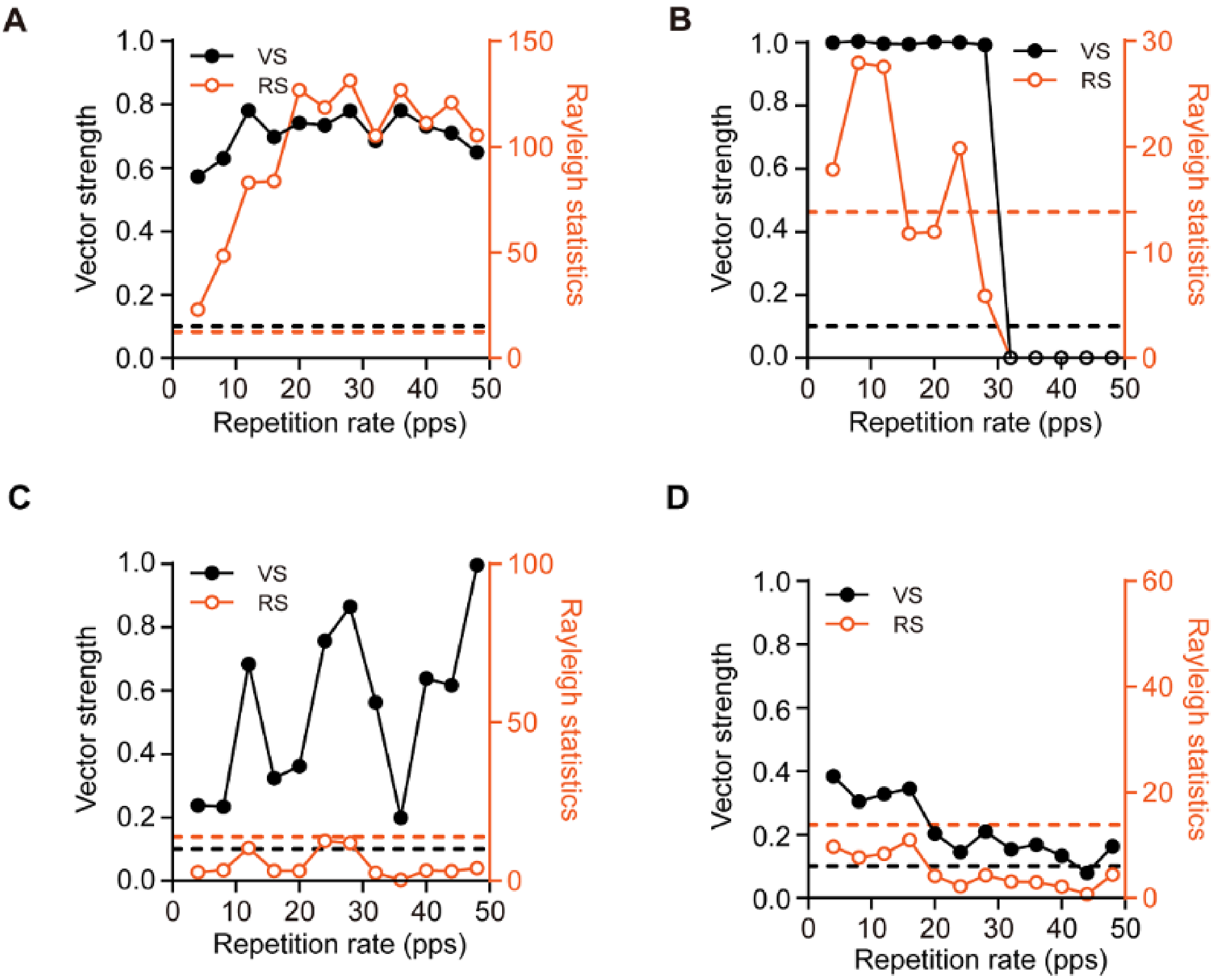


**Fig. S1** Stimulus synchronized and non-synchronized IC neurons. **A–D,** Vector strength (VS, black) and Rayleigh statistics (RS, orange) of the syn+ (**A**), syn− (**B**), nonsyn+ (**C**) and nonsyn responses (**D**) IC neurons in response to Gaussian click trains with varying repetition rates at the flutter range. Horizontal black dashed line at 0.1 (related to left y axis) indicates minimal VS for stimulus-synchronized response; Horizontal orange dashed line at 13.8 (related to right y axis) indicates significance in RS, which is equal to $P < 0.001$.

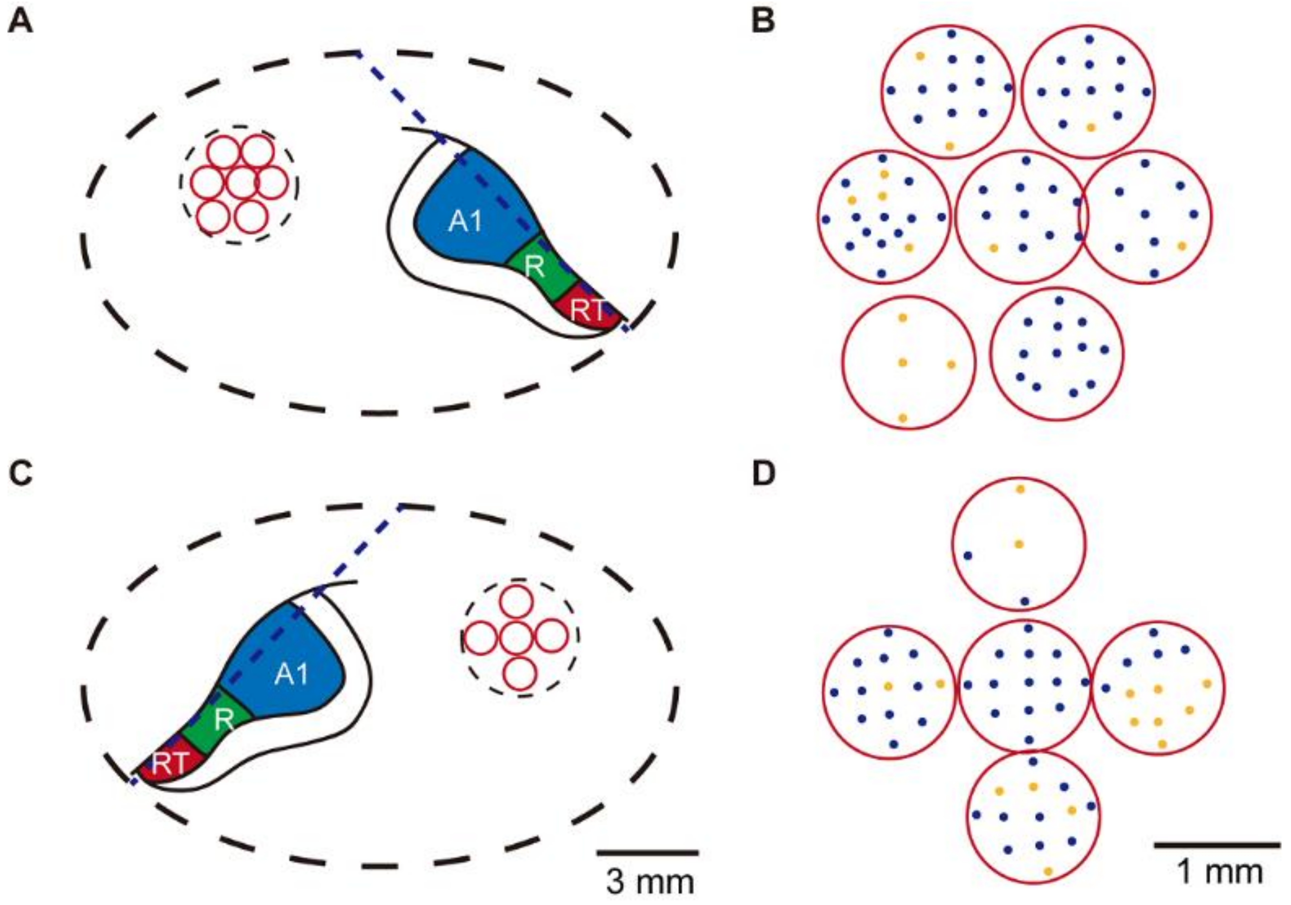


**Fig. S2** Recording sites in the inferior colliculus of marmosets. **A–B** Recording sites in IC of the right hemisphere of a marmoset (ID: 180512E). **A** lateral view of the recording chamber. The dashed oval indicates the chamber built during head-cap implant surgery. Location of the primary auditory cortex (A1), Rostral area (R), Rostrotemporal area (RT), and IC (dashed gray circle) was shown in the figure. The red circles are 1 mm holes drilled on the skull for electrode penetrations, which were expanded in B. **B** Electrode penetrations made in the recording holes. Blue dots, penetrations with significant auditory responses in IC; yellow dots, penetrations without auditory responses in IC. **C–D** Recording sites in IC of the left hemisphere of a marmoset (ID: JSF5). The illustration of C–D was identical to A and B.
